# Abnormal Spontaneous Gamma Power Is Associated With Verbal Learning and Memory Dysfunction in Schizophrenia

**DOI:** 10.3389/fpsyt.2020.00832

**Published:** 2020-08-31

**Authors:** Kumiko Tanaka-Koshiyama, Daisuke Koshiyama, Makoto Miyakoshi, Yash B. Joshi, Juan L. Molina, Joyce Sprock, David L. Braff, Gregory A. Light

**Affiliations:** ^1^Department of Psychiatry, University of California San Diego, La Jolla, CA, United States; ^2^Division of Law and Psychiatry, Center for Forensic Mental Health, Graduate School of Medical and Pharmaceutical Sciences, Chiba University, Chiba, Japan; ^3^Department of Psychiatry, Tokyo Metropolitan Matsuzawa Hospital, Tokyo, Japan; ^4^Swartz Center for Neural Computation, University of California San Diego, La Jolla, CA, United States; ^5^VISN-22 Mental Illness, Research, Education and Clinical Center (MIRECC), VA San Diego Healthcare System, San Diego, CA, United States

**Keywords:** spontaneous gamma oscillation, resting-state electroencephalography (EEG), schizophrenia, cognitive function, memory, verbal learning, translational biomarker

## Abstract

**Background:**

Schizophrenia patients exhibit cognitive deficits across multiple domains, including verbal memory, working memory, and executive function, which substantially contribute to psychosocial disability. Gamma oscillations are associated with a wide range of cognitive operations, and are important for cortico-cortical transmission and the integration of information across neural networks. While previous reports have shown that schizophrenia patients have selective impairments in the ability to support gamma oscillations in response to 40-Hz auditory stimulation, it is unclear if patients show abnormalities in gamma power at rest, or whether resting-state activity in other frequency bands is associated with cognitive functioning in schizophrenia patients.

**Methods:**

Resting-state electroencephalogram (EEG) was assessed over 3 min in 145 healthy comparison subjects and 157 schizophrenia patients. Single-word reading ability was measured *via* the reading subtest of the Wide Range Achievement Test-3 (WRAT). Auditory attention and working memory were evaluated using Letter-Number Span and Letter-Number Sequencing. Executive function was assessed *via* perseverative responses on the Wisconsin Card Sorting Test (WCST). Verbal learning performance was measured using the California Verbal Learning Test second edition (CVLT-II).

**Results:**

Schizophrenia patients showed normal levels of delta-band power but abnormally elevated EEG power in theta, alpha, beta, and gamma bands. An exploratory correlation analysis showed a significant negative correlation of gamma-band power and verbal learning performance in schizophrenia patients.

**Conclusions:**

Patients with schizophrenia have abnormal resting-state EEG power across multiple frequency bands; gamma-band abnormalities were selectively and negatively associated with impairments in verbal learning. Resting-state gamma-band EEG power may be useful for understanding the pathophysiology of cognitive dysfunction and developing novel therapeutics in schizophrenia patients.

## Introduction

Schizophrenia patients demonstrate cognitive deficits across multiple domains that directly contribute to psychosocial disability (1). Since verbal memory is a robust determinant of functional outcomes (2), biomarkers that are linked to the targeted domains are awaited, for a better understanding of pathological conditions and for creating animal models for therapeutic development.

Among the many neurophysiological abnormalities seen in schizophrenia, gamma-band oscillatory deficits have been consistently reported (3–5) and are thought to underlie cognitive deficits (6). Gamma oscillations (i.e., above 30 Hz) are associated with a wide range of cognitive operations through cortico-cortical transmission and the integration of information across neural networks in healthy subjects (7–13). Gamma-band activity can be measured *via* variety of analytic and experimental settings (e.g., spontaneous, evoked, induced, and emitted) and across different modalities (electroencephalography, EEG; magnetoencephalography, MEG), which have led to the identification of gamma-band abnormalities in a broad range of experimental contexts in schizophrenia patients (3, 4, 14). Associations of cognitive dysfunction and evoked or induced gamma-band activity in schizophrenia patients are well known (6, 15, 16). However, the association of cognitive deficits and spontaneous gamma oscillation has been relatively less studied.

Spontaneous gamma-band power obtained by resting-state EEG may be useful in understanding cognitive dysfunction in schizophrenia patients. Resting-state EEG, which is spontaneous and does not utilize a task, has already been widely implemented in clinical settings. A recent review article of resting-state EEG in schizophrenia patients found differences in eyes-closed vs. eyes-open recording conditions (17). In recordings with the eyes closed, schizophrenia patients showed increases in the absolute delta- and theta-band power and decreases in the alpha-band power with no difference in the beta-band power compared to healthy subjects (17). In eyes-open conditions, the patients showed significantly increased in theta-, alpha-, and beta-band power with no difference in the delta-band power compared to healthy subjects (17). Notably, gamma-band power was not reported. While some studies showed increased resting-state gamma-band power in schizophrenia in eyes-closed condition (18–20), Grent-’t-Jong et al. (21) found an increased gamma power (30–46 Hz) at the occipital cortex and a decreased at the prefrontal cortex in first episode schizophrenia patients (N = 21) and showed a decreased MEG gamma power at the frontal, temporal, and sensorimotor cortices in chronic schizophrenia patients (N = 34) compared to healthy controls (N = 37). However, the specificity of frequency band abnormalities and their associations with cognitive dysfunction in schizophrenia are largely unknown.

We hypothesized that schizophrenia patients would show significantly increased power across multiple frequency bands, including gamma. Given the many studies that have demonstrated the importance of evoked or induced gamma oscillations in supporting cognition (6, 15, 16), we hypothesized that spontaneous gamma power would be also associated with cognitive functioning in schizophrenia. In the current study, resting-state EEG absolute power across multiple frequency bands (delta, theta, alpha, beta, and gamma) was assessed in healthy comparison subjects and schizophrenia patients with open eyes. Eyes-open condition approaches to the real-world environment in which cognitive functions actually work, in addition to preventing subjects from falling asleep. Exploratory correlational analyses between EEG power and cognitive function, such as intellectual/verbal ability, attention, working memory, executive function, verbal learning and memory performance were also conducted.

## Materials and Methods

### Subjects

Participants included 145 healthy comparison subjects and 157 schizophrenia patients. Patients were recruited from community residential facilities and *via* clinician referral and diagnosed using a modified version of the structured clinical interview for DSM-IV-TR. Antipsychotics, anxiolytics, and anticholinergics were prescribed for 140, 25, and 47 schizophrenia patients, respectively. Healthy comparison subjects were recruited through internet advertisements. Healthy comparison subjects who reported any psychopathology, past treatment for a psychiatric disorder including hospitalization or electroconvulsive therapy (ECT), or having been treated with any psychoactive medications in the past or currently were excluded. In addition, those who reported having a first degree relative with schizophrenia or any other psychotic disorder were excluded. Exclusion criteria for all subjects included an inability to understand the consent processes and/or provide consent or assent, not being a fluent English speaker, previous significant head injury with loss of consciousness, significant substance abuse during the prior 6 months, neurologic illness, or severe systemic illness. Moreover, all subjects received confidential urine toxicology screens for drugs of abuse and were excluded if the test was positive. Written informed consent was obtained from each subject. The Institutional Review Board of University of California San Diego approved all experimental procedures (071831 and 170147). We assessed clinical symptoms in the schizophrenia patients with the Scale for the Assessment of Negative Symptoms (SANS; scores ranged from 0–25, with higher scores indicating severe symptom) (22) and the Scale for the Assessment of Positive Symptoms (SAPS; scores ranged from 0–20, with higher scores indicating severe symptom) (23). We rated functional outcomes using the Global Assessment of Functioning scale (GAF). The GAF evaluates the overall level of social adaptation from 0 to 100 scores. A higher score means a higher function.

### Neuropsychological Measures

Since cognitive dysfunction such as intelligence, attention, working memory, executive functioning, and verbal learning have been reported to be associated with functional outcomes in schizophrenia patients (1, 2, 24–26), we assessed those abilities in the current study. Single-word reading ability was measured *via* the Reading subtest of the Wide Range Achievement Test-3 (WRAT) (27) to estimate premorbid verbal functioning. Raw scores were converted to standard scores, which range from 55 to 145 and have a mean of 100 and a standard deviation (SD) of 15, with higher scores indicating greater ability. Auditory attention and working memory were evaluated using Letter-Number Span and Letter-Number Sequencing with higher scores indicating greater ability (28). The numbers of perseverative responses obtained in the Wisconsin Card Sorting Test (WCST) was used to assess executive functioning, with lower scores indicating greater ability (29, 30). Verbal learning and memory performance were assessed *via* the California Verbal Learning Test second edition (CVLT-II) using total correct scores from the Total Learning (list A trials 1–5), with higher scores indicating greater ability (31).

### Electroencephalography Recording and Preprocessing

During the session, participants sat in a comfortable chair in a quiet room and were instructed to relax and watch a silent cartoon video with their eyes open. Subjects were closely monitored to ensure that subjects remained awake.

EEG data were continuously digitized at a rate of 1,000 Hz (nose reference and forehead ground) using a 40-channel Neuroscan system (Neuroscan Laboratories, El Paso, Texas). The electrode montage was based on standard positions in the International 10–5 electrode system (32) fit to the Montreal Neurological Institute (MNI) template head used in EEGLAB 14.1.2 (33) and included AFp10 and AFp9 as horizontal EOG channels, IEOG and SEOG above and below the left eye as vertical EOG channels, Fp1, Fp2, F7, F8, Fz, F3, F4, FC1, FC2, FC5, FC6, C3, Cz, C4, CP1, CP2, CP5, CP6, P7, P3, Pz, P4, P8, T7, T8, TP9, TP10, FT9, FT10, PO9, PO10, O1, O2, and Iz. Electrode-to-skin impedance mediated by conductive gel was maintained below 4 kΩ. The system acquisition bandpass was 0.5–100 Hz. Offline, EEG data were imported to EEGLAB running under MATLAB 2017b (The MathWorks, Natick, MA). Data were high-pass filtered [finite impulse response (FIR), Hamming window, cutoff frequency of 0.5 Hz, and transition bandwidth of 0.5]. EEGLAB plugin *clean_rawdata()* including artifact subspace reconstruction (ASR) was applied to reduce high-amplitude artifacts (34–36). The parameters used were as follows: flat line removal, 10 s; electrode correlation, 0.7; ASR, 20; and window rejection, 0.5. The mean final data length was 328 s (SD, 82; range, 203–1,555]. The mean final data length was not significantly different between healthy comparison subjects (mean, 324; SD, 41; range, 278–588) and schizophrenia patients (mean, 331; SD, 108; range, 203–1,555; *t* = 0.68, *p* = 0.50). Mean channel rejection rate was 4.4% (SD, 3.4; range, 0–18.4). Mean data rejection rate was 2.3% (SD, 3.8; range, 0–36.1). The rejected channels were interpolated using EEGLAB’s spline interpolation function. Data were re-referenced to average. Adaptive mixture independent component analysis (ICA) (37) was applied to the preprocessed scalp recording data to remove ocular artifacts and obtain temporally maximally independent components (ICs). For scalp topography of each independent component derived, equivalent current dipole was estimated using Fieldtrip functions (38). For scalp topographies more suitable for symmetrical bilateral dipoles, two symmetrical dipoles were estimated (39). To select brain ICs among all types of ICs, EEGLAB plugin *ICLabel()* was used (40). The inclusion criteria were 1) ‘brain’ label probability > 0.7 and 2) residual variance, i.e., var((actual scalp topography) – (theoretical scalp projection from the fitted dipole))/var(actual scalp topography) < 0.1.

### Statistical Analysis

All statistical analyses were conducted using SPSS version 26.0 (IBM Corp., Armonk, NY). We used χ^2^ tests and independent *t*-tests to compare the demographic data between the groups, and we applied a statistical significance threshold of *p* < 0.05. For comparisons of neuropsychological measures between healthy comparison subjects and patients, we used independent *t*-test and applied a significance threshold of *p* < 0.01 (0.05/5; five neurocognitive measures) adjusted with Bonferroni correction; Cohen’s *d* effect sizes were calculated from the overall group contrast.

To get an overall impression, power spectrum was calculated for grand mean across 34 electrodes in healthy subjects and schizophrenia patients, respectively. Furthermore, we compared the power for each 0.1 Hz frequency between the groups applying to statistical significance *p* < 0.05 false discovery rate corrected.

Spatial principal component analysis (PCA) was performed with EEG band power of 34 electrodes in each of the five bands in 302 subjects to reduce redundant scalp information and extract representative values in accordance with a previous study (41). We set the five bands as delta (1–3 Hz), theta (4–7 Hz), alpha (8–13 Hz), beta (14–29 Hz), and gamma (30–50 Hz) (42).

A repeated-measures analysis of covariance (ANCOVA) with EEG band power (delta, theta, alpha, beta, and gamma) as the within-subject factor, group (healthy comparison and schizophrenia groups) as the between-subjects factor, and age, sex, and education as the covariates was performed. Greenhouse-Geisser corrections were reported when Mauchly’s test for sphericity was significant. Statistical significance was set at *p* < 0.05. If a main effect of the group was identified, then *post hoc* ANCOVA with age, sex, and education as the covariates was performed for each EEG band power (statistical significance was set at *p* < 0.05, and false discovery rate was corrected).

Multiple regression with the backward elimination was applied to frequency band and cognitive measures; standardized beta coefficients are presented below.

$$(\text{Neurocognitive measures})=\beta_{0}+\beta_{1}\times (\text{theta power})+\beta_{2}\times (\text{alpha power})+\beta_{3}\times (\text{beta power})+\beta_{4}\times (\text{gamma power})+\epsilon$$

Statistical significance was set at *p* < 0.01 (0.05/5; five neurocognitive measures) adjusted with Bonferroni correction. Since anxiolytics and anticholinergics medications are known to impact resting-state EEG frequency band parameters and/or cognition (43–45), regression analyses were performed in all patients and after removing a subset of patients prescribed either anxiolytics or anticholinergics medications at the time of testing (N = 93).

For supplementary information, these same models were applied to show the association between clinical symptoms (positive symptoms, negative symptoms and functional outcomes) and EEG band power. Statistical significance was set at *p* < 0.017 (0.05/3; three clinical symptom measures) adjusted with Bonferroni correction.

Finally, *post hoc* multiple regression with the backward elimination methods was explored with gamma power at 34 channels to determine whether regional oscillatory dynamics were associated with verbal memory impairments.

$$(\text{CVLT scores})=\beta_{0}+\beta_{1}\times (\text{Fp}1\text{ gamma power})+\beta_{2}\times (\text{Fp}2\text{ gamma power})+\beta_{3}\times (\text{Fp}7\text{ gamma power})+\ldots +\beta_{34}\times (\text{Iz gamma power})+\epsilon$$

Statistical significance was set at *p* < 0.01 (0.05/5; five neurocognitive measures) adjusted with Bonferroni correction.

## Results

### Demographics

The mean (SD) age of the 145 healthy comparison subjects was 39.9 (12.8) years old; education duration was 14.7 (2.1) years (**Table 1**). The mean age of the 157 patients was 46.4 (10.8) years old; education duration was 12.4 (2.1) years; duration of illness was 25.1 (11.9) years; SAPS total average score was 6.8 (4.0); SANS total score was 16.9 (3.8); GAF average score was 41.1 (4.3). The mean age was significantly higher in patients compared to healthy subjects (*t* = 4.8, *p* = 2.7 × 10^–6^); the education duration was significantly lower in patients compared to healthy subjects (*t* = –9.0, *p* = 3.8 × 10^–17^). The sex ratio (male/female) was significantly different between the groups (healthy subjects, 68/77; schizophrenia patients, 103/54; *χ*^2^ = 10.7, *p* = 1.0 × 10^–3^).

**Table 1:** Demographic data of subjects.

	Healthy comparison subjects	Schizophrenia patients	Statistics
Gender (Male/Female)	68/77	103/54	*χ*^2^ = 10.7, *p* = 1.0 × 10^–3^*
Age (year)	39.9 (12.8)	46.4 (10.8)	*t* = 4.8, *p* = 2.7 × 10^–6^*
Education (year)	14.7 (2.1)	12.4 (2.1)	*t* = –9.0, *p* = 3.8 × 10^–17^*
Duration of illness (year)^a^		25.1 (11.9)	
SAPS^b^		6.8 (4.0)	
SANS^b^		16.9 (3.8)	
GAF		41.1 (4.3)	

Asterisks indicate statistical significance p < 0.05; ^a^Two subjects have no data of duration of illness; ^b^One subjects have no data of SAPS and SANS.

SAPS, Scale for the Assessment of Positive Symptoms; SANS, Scale for the Assessment of Negative Symptoms; GAF, Global Assessment of Functioning.

### Differences in Neurocognitive Measures Between in Healthy Comparison Subjects and in Schizophrenia Patients

As shown in **Table 2**, schizophrenia patients showed significant impairments across all cognitive domains.

**Table 2:** Comparing of cognitive function between healthy comparison subjects and schizophrenia patients.

	Healthy comparison subjects	Schizophrenia patients	Effect size	Statistics
	(N = 145)	(N = 157)		
WRAT^a^	101.8 (10.6)	94.9 (13.0)	–0.58	*t* = –4.9, *p* = 1.3 × 10^–6*^
LN span^b^	12.8 (3.0)	10.9 (2.5)	–0.73	*t* = –6.3, *p* = 9.9 × 10^–10*^
LN sequencing^b^	10.0 (2.4)	7.7 (2.4)	–0.95	*t* = –8.3, *p* = 4.9 × 10^–15*^
WCST^c^	8.5 (7.2)	17.7 (14.9)	0.79	*t* = 6.7, *p* = 1.2 × 10^–10*^
CVLT^d^	51.0 (10.0)	40.9 (11.8)	–0.92	*t* = –7.9, *p* = 3.9 × 10^–14*^

Asterisks indicate statistical significance p < 0.01 (0.05/5; five neurocognitive measures) adjusted with Bonferroni correction; Effect size is Cohen’s d; ^a^Three healthy comparison subjects and five schizophrenia patients have no data of WRAT; ^b^One healthy comparison subject has no data of LN span and LN sequencing; ^c^Four healthy comparison subjects and one schizophrenia patient have no data of WCST; ^d^Two healthy comparison subject have no data of CVLT.

WRAT, Wide Range Achievement Test; LN, letter-number, WCST; Wisconsin Card Sorting Test; CVLT, California Verbal Learning Test.

### Electroencephalographic Data

**Figure 1:**
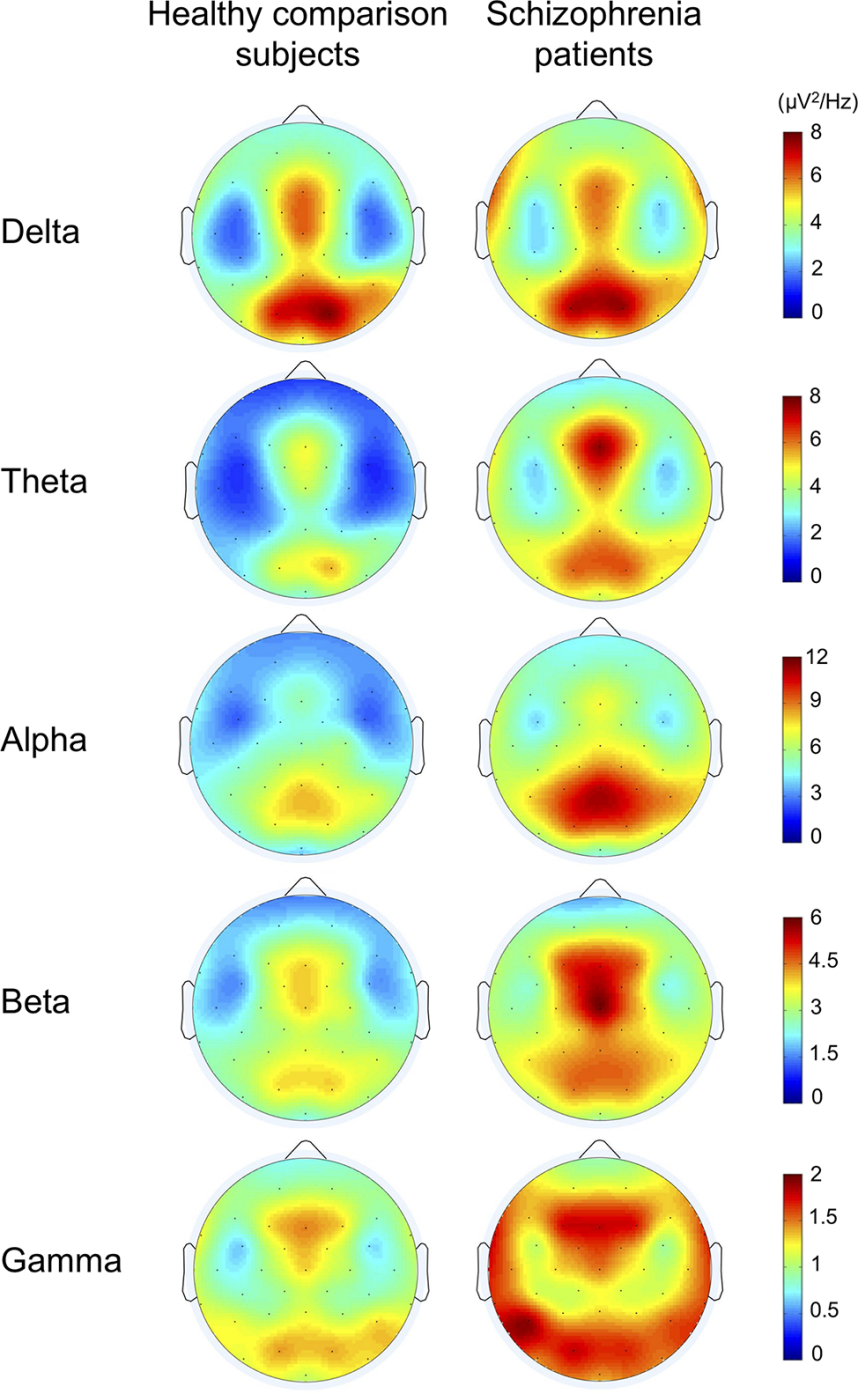
The topographies for grand average spectral power in healthy comparison subjects and in schizophrenia patients.

**Figure 2:**
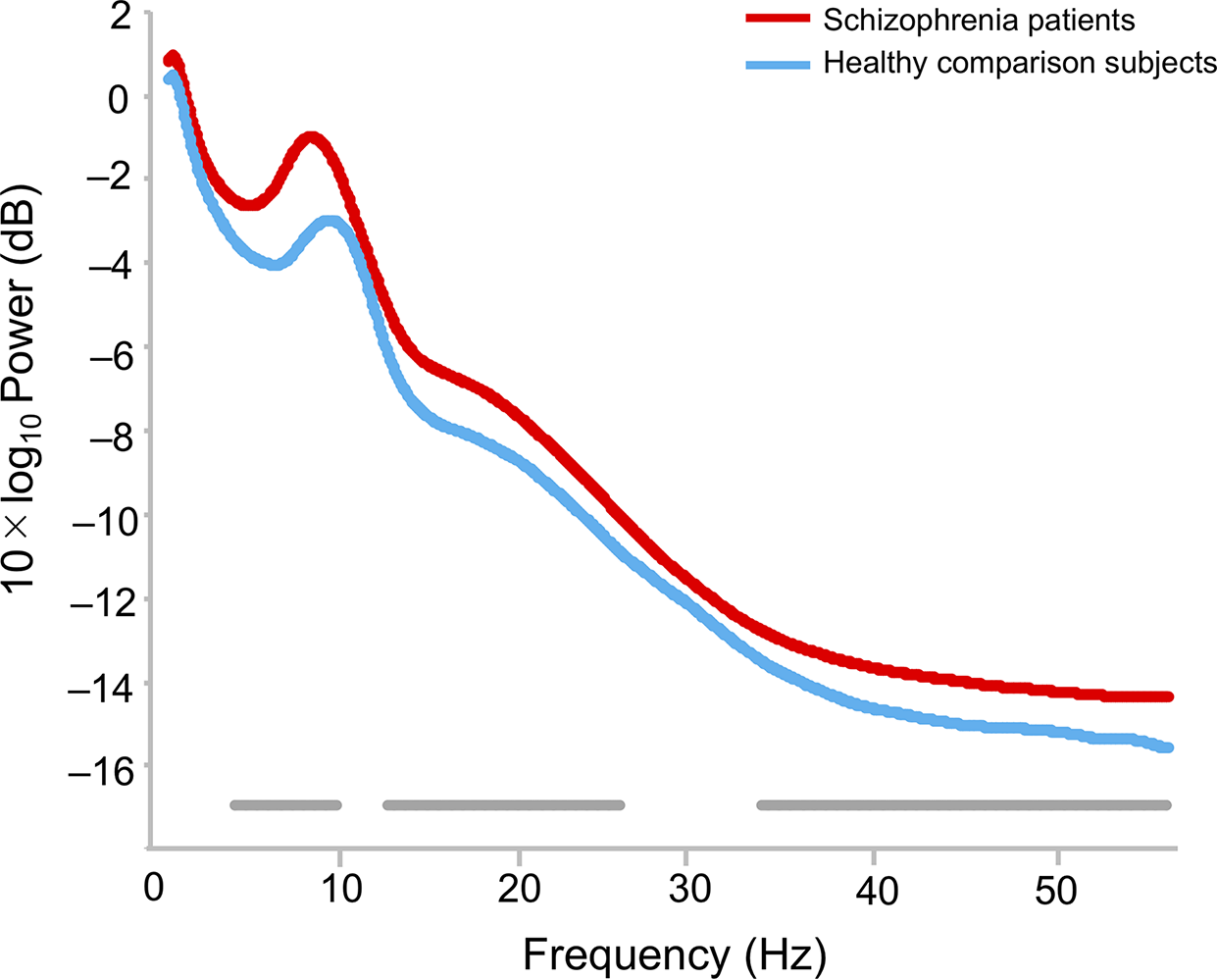
Difference of power spectrum in healthy comparison subjects and schizophrenia patients. The bottom gray bars indicate statistical significance *p* < 0.05 (false discovery rate corrected).

The topographies for grand average spectral power (μV^2^/Hz) are shown in **Figure 1**. Grand mean power spectrum is shown in **Figure 2**. The power spectrum showed significant higher power in theta, alpha, beta, and gamma bands in schizophrenia patients compared to healthy subjects. PCA showed that the 1st component of delta power was 80.7%; 2nd component of delta power, 6.2%; 3rd component of delta power, 3.2%; 1st theta, 84.2%; 2nd theta, 3.7%; 1st alpha, 81.4%; 2nd alpha, 4.5%; 3rd alpha, 3.2%; 1st beta, 76.1%; 2nd beta, 5.9%; 3rd beta, 3.4%; 1st gamma, 58.7%; 2nd gamma, 6.9%; 3rd gamma 4.2%; 4th gamma 4.2%; 5th gamma 3.4%. The first principal component that accounted for the largest proportion of variance was selected for subsequent analyses. The proportion of variance in each band power was comparable to findings in healthy subjects and patients with depressive disorder recently reported by Koshiyama et al. (41).

### EEG Band Power Difference Between in Healthy Comparison Subjects and in Schizophrenia Patients

**Figure 3:**
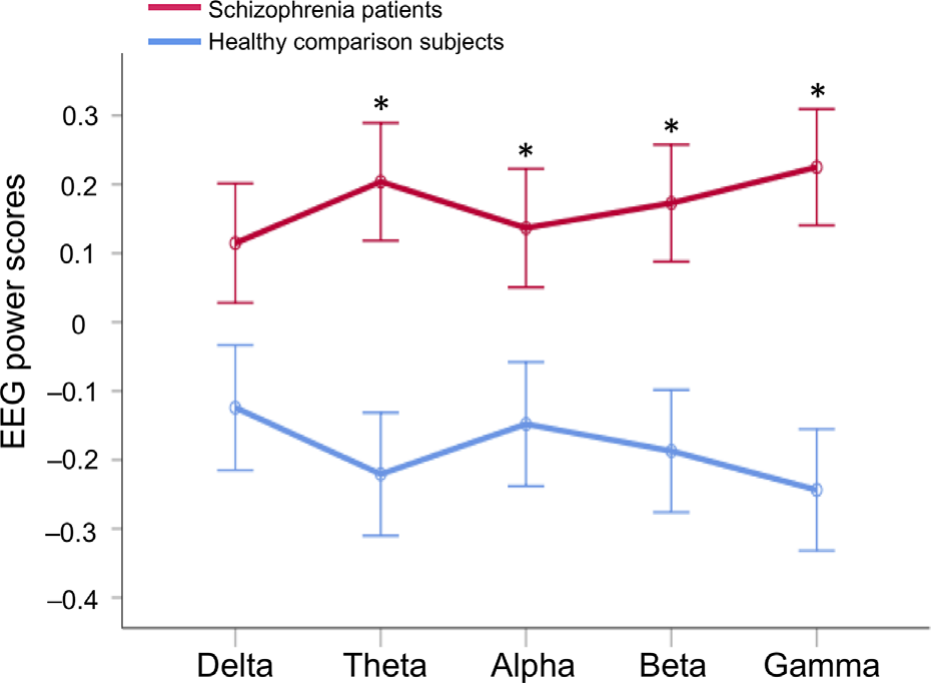
EEG band power difference between in healthy comparison subjects and in schizophrenia patients. A repeated-measures analysis of covariance (ANCOVA) was performed after adjustment for age, sex, and education; Asterisks indicate statistical significance *p* < 0.05 (false discovery rate corrected) with *post hoc* ANCOVA after adjustment for age, sex, and education to compare the groups in each EEG band power. EEG, electroencephalography.

Repeated-measures ANCOVA showed a significant main effect of the group (*F*_1, 297_ = 9.5, *p* = 2.2 × 10^–3^), but no main effect of the EEG band power (*F*_2.5, 751.0_ = 1.1, *p* = 0.34) nor interaction between the group and EEG band power after adjustment for age, sex, and education (*F*_2.5, 751.0_ = 1.6, *p* = 0.20; **Figure 3**). *Post hoc* ANCOVA for each band power revealed significant differences between groups in theta (*F*_1, 297_ = 10.2, *p* = 1.6 × 10^–3^), alpha (*F*_1, 297_ = 4.5, *p* = 0.035), beta (*F*_1, 297_ = 7.4, *p* = 6.9 × 10^–3^), and gamma (*F*_1, 297_ = 12.7, *p* = 4.3 × 10^–4^), but not in delta power (*F*_1, 297_ = 3.1, *p* = 0.08) after adjustment for age, sex, and education, respectively.

### Exploratory Correlation Analyses Between Neurocognitive Measures and EEG Band Power in Schizophrenia Patients

With the backward elimination method, multiple regression analyses yielded one regressor of the component score for gamma-band power on CVLT score in the patients (*β* = –0.32, *p* = 3.7 × 10^–5^; **Figure 4**; analysis of variance for regression, *F*_1, 155_ = 18.1, *p* < 0.001; adjusted *R^2^* = 0.10). No other significant relationships of EEG frequency bands with clinical, cognitive, or functional measures were observed.

**Figure:**
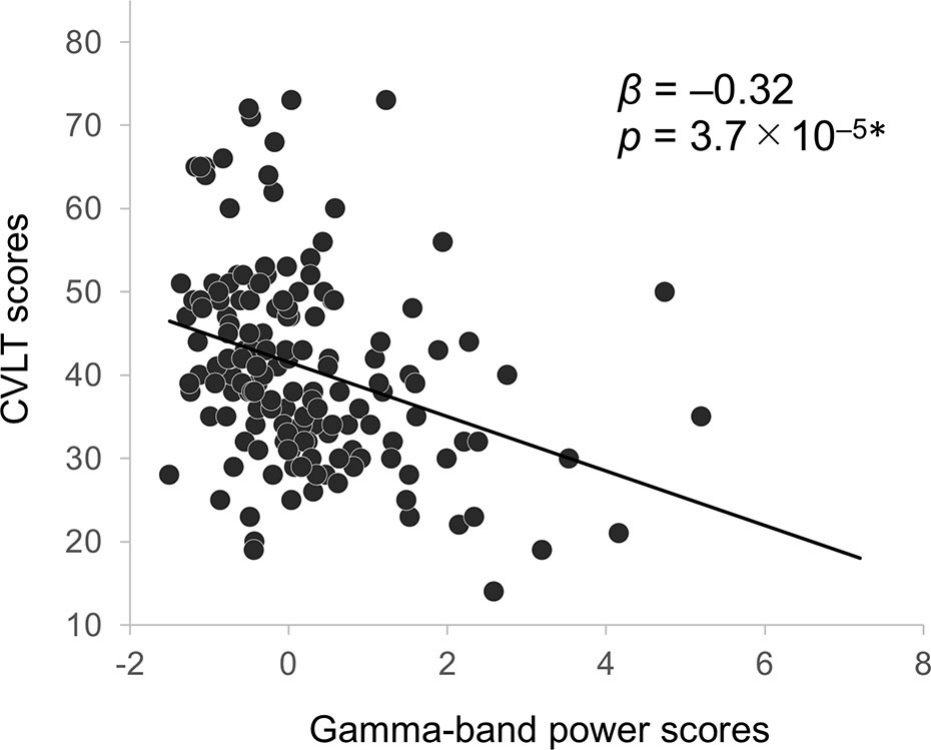
**Figure 4** Correlation between CVLT scores and gamma-band EEG power in schizophrenia patients. Asterisks indicate statistical significance *p* < 0.01 (0.05/5; five neurocognitive measures) adjusted with Bonferroni correction. CVLT, California Verbal Learning Test; EEG, electroencephalography.

We confirmed the associations of gamma power and verbal learning were comparable when analyses were restricted to the subset of patients who were not prescribed either anxiolytics or anticholinergics medications at the time of testing (*β* = –0.38, *p* = 1.7 × 10^–4^; N = 93; regression analysis with the backward elimination method; analysis of variance for regression, *F*_1, 91_ = 15.4, *p* < 0.001; adjusted *R^2^* = 0.14).

*Post hoc* regression analyses revealed significant associations of gamma-band power with CVLT performance (*F*_5, 151_ = 11.1, *p* < 0.001; adjusted *R^2^* = 0.24) at Fp2 (*β* = –0.52, *p* = 9.4 × 10^–6^), at F4 (*β* = 0.28, *p* = 4.6 × 10^–3^), and at T8 (*β* = 0.27, *p* = 6.6 × 10^–3^).

## Discussion

In the current study, schizophrenia patients showed significantly elevated EEG power in theta, alpha, beta, and gamma frequency bands; the abnormally elevated gamma-band power was negatively correlated with verbal learning performance in schizophrenia patients. While verbal learning ability was significantly negatively correlated with gamma-band activity at Fp2, verbal learning ability was positively correlated with gamma-band activity at F4 and T8.

The analysis by using PCA showed a general increase in theta-, alpha-, beta-, and gamma-bands power. Although we cannot directly compare the previous results shown by power spectral density with the current results shown by PCA, the direction in which increases are shown from theta through gamma bands in the current study is consistent to the previous studies with eyes-open conditions (17, 46–48). Relevant to gamma-band power, while a previous resting-state MEG study with eyes-open condition by Grent-’t-Jong et al. (21) showed an increased gamma power (30–46 Hz) at the occipital cortex and a decreased at the prefrontal cortex in first episode schizophrenia patients (N = 21), they demonstrated a decreased gamma power at the frontal, temporal, and sensorimotor cortices in chronic schizophrenia patients (N = 34) compared to healthy controls (N = 37). Inconsistency of their results (21) and our findings of elevated gamma-band power in chronic schizophrenia patients may be due to the difference of clinical stages. Mean age of their chronic schizophrenia patients was 37.1 years old, while that of our subjects was 46.4 years old.

In the current study, resting-state gamma-band abnormalities were associated with verbal learning impairment in schizophrenia patients. Interestingly, resting-state EEG power was not associated with measures of single word reading, auditory attention, working memory, or executive functioning. Topographical distributions (**Figure 1**) revealed that the higher gamma oscillatory power was most robust in the frontal region, although temporal-occipital dysfunction is also prominent. Indeed, we found specific regional gamma associations with verbal memory impairments at three right front-temporal electrodes. The finding of aberrant gamma power over frontal regions suggests that this higher frequency “cortical noise” may be a contributor to verbal learning deficits in schizophrenia patients. Evidence for structural alterations in frontal regions related to cognitive functions in schizophrenia are widespread (49–51). Similarly, evidence for structural alterations have been correlated with abnormal gamma oscillations (52, 53). Furthermore, some investigators detected alterations of gamma-band oscillation in response to 40-Hz stimuli at Fz correlated with cognitive dysfunction and lower global functioning in schizophrenia patients; our findings are consistent with those findings (54–56).

We should note some limitations of this study. First, study participants included patients with well-established chronic illness; results may not generalize to younger or early-illness cohorts. Second, as in many cross-sectional studies of patients with chronic psychosis, medications were not experimentally controlled. Carefully controlled longitudinal studies are needed to assess the acute and chronic effects of medications. In this context, although anxiolytics and anticholinergics medications are known to impact resting-state EEG frequency band parameters and/or cognition (43–45), we confirmed that associations of gamma power and verbal learning were comparable when analyses were restricted to the subset of patients who were not prescribed either class of medication at the time of testing. Third, the present results assessed global EEG power derived from a PCA-based approach to scalp channel data to extract the characteristics of each band. While the first components accounted for the vast majority of variance in each band over all scalp electrodes, they did not fully explain all of the variance. However, the maximum components other than the first components did not exceed 7%, and the effects were considered to be limited. While conventional approaches to resting state EEG have assessed effects at pre-selected individual electrodes or region of interest (ROI)-based clusters of electrodes, the PCA method used in this paper provides a data-driven approach for characterizing macroscale/global oscillatory effects. Fourth, although the proportion of variance of the first principal component in gamma-band power was relatively low (58.7%), it was consistent with a previous study (41); this may be due to the relatively small amplitude of gamma oscillations compared to other frequency bands. Slow waves are associated with cortico-cortical communications over relatively long distances *via* structures such as white matter fasciculus, but gamma waves derive from comparatively local neural network activity. Although speculative, locations of smaller local networks may spatially vary in individuals compared to those of macro networks *via* large structures. Lastly, findings of resting-state gamma abnormalities may not generalize to more commonly studied stimulus-driven forms of gamma-band responses (54, 57–59). Future studies are needed to assess the relationships among spontaneous gamma activity at rest with gamma oscillations that are generated in response to 40-Hz stimulation.

In conclusion, patients with schizophrenia show abnormal resting-state EEG power across multiple frequency bands. Abnormalities in spontaneous gamma activity were selectively associated with impairments in verbal learning for the first time. Resting-state gamma-band EEG power may be a useful biomarker for understanding the pathophysiology of cognitive dysfunction in schizophrenia patients and developing novel therapeutics.

## Data Availability Statement

The datasets presented in this article are not readily available because: Due to ethical issues, patients’ data cannot be disclosed to third parties. Requests to access the datasets should be directed to GL, glight@health.ucsd.edu.

## Ethics Statement

The studies involving human participants were reviewed and approved by Institutional Review Board of University of California San Diego. The patients/participants provided their written informed consent to participate in this study. Written informed consent was obtained from the individual(s) for the publication of any potentially identifiable images or data included in this article.

## Author Contributions

JS, DB, and GL collected the data. KT-K, DK, and MM analyzed the data. KT-K, DK, MM, YJ, JM, DB, and GL interpreted the results. KT-K, DK, and GL designed the study. GL supervised all aspects of collection, analysis, and interpretation of the data. KT-K, DK, MM, and GL wrote the original manuscript. YJ, JM, JS, and DB reviewed and edited the manuscript. All authors contributed to the article and approved the submitted version.

## Funding

This study was supported by the JSPS Overseas Research Fellowships (DK), the Sidney R. Baer, Jr Foundation, and the VISN-22 Mental Illness Research Education and Clinical Center. Swartz Center for Computational Neuroscience is supported by the generous gift of Swartz Foundation (New York). The funders had no role in the study design, data collection and analysis, publication decision, or manuscript preparation.

## Conflict of Interest

The authors declare that the research was conducted in the absence of any commercial or financial relationships that could be construed as a potential conflict of interest.
